# Comparison of sperm selection techniques in donkeys: motile subpopulations from a practical point of view

**DOI:** 10.21451/1984-3143-AR2018-0133

**Published:** 2019-10-23

**Authors:** Isabel Ortiz, Jesús Dorado, Jane M. Morrell, Maria Angeles Diaz-Jimenez, Blasa Pereira, César Consuegra, Manuel Hidalgo

**Affiliations:** 1 Veterinary Reproduction Group, Department of Medicine and Animal Surgery, Faculty of Veterinary Medicine, University of Cordoba, Cordoba, Spain.; 2 Department of Clinical Sciences, Division of Reproduction, Swedish University of Agricultural Sciences, Uppsala, Sweden.

**Keywords:** cryopreservation, SLC, subpopulations

## Abstract

The aim of this study was to compare the post-thaw distribution of motile sperm subpopulations, following simple or colloid centrifugation. A new analysis was used to evaluate the available number of sperm from each subpopulation after each centrifugation protocol. Frozen/thawed semen samples were divided into the following after-thawing treatments: uncentrifuged control (UDC), sperm washing (SW) and two colloid centrifugation procedures (Equipure, SLC-E, and Androcoll, SLC-A). Percentage of total and progressive motility (TM and PM), as well as sperm motility kinematics, distribution of motile sperm subpopulations, and recovery rates, were statistically compared among treatments. The SLC treatments showed higher (P < 0.001) TM and PM than UDC and SW. Following each SLC procedure, different percentages of the subpopulation with the most vigorous and progressive sperm (sP4) were obtained. SLC-A recovered a larger number of sperm belonging to sP4 than SLC-E, but not significantly higher than SW. From a practical point of view, sperm washing, the standard centrifugation procedure for equine semen processing, recovered the same amount of fast and progressive sperm as colloid centrifugation, apparently the best treatment according to traditional analysis. In conclusion, samples processed by SLC have higher motility percentages than SW and UDC but, after combining the available number of sperm, SLC and SW techniques are equally efficient in recovering sperm from the most vigorous, fast and progressive motile subpopulation (sP4).

## Introduction

Freezing and thawing cause major damage to stallion and donkey sperm, mainly due to osmotic stress and penetrant cryoprotectants (Peña *et al*., 2011). Treatments such as sperm washing and colloid centrifugation have been proposed as a strategy to improve post-thaw sperm quality (Ortiz *et al*., 2015a) and fertility of donkey spermatozoa (Serres *et al*., 2014). These procedures have been shown to select sperm mainly in terms of sperm motility (Ortiz *et al*., 2015b). This parameter plays an essential role in fertility, allowing sperm to travel along the female reproductive tract and penetrate the oocyte (Colenbrander *et al*., 2003). Recently, different sperm motility subpopulations has been found in the ejaculates of several animal species (Martinez-Pastor *et al*., 2005; Flores *et al*., 2008; Muiño *et al*., 2009; Ortega-Ferrusola *et al*., 2009; Dorado *et al*., 2010). Motile subpopulations are relatively homogeneous subgroups of sperm with well-defined kinematic features in each species and for instance, have been related to fertility in donkeys (Dorado *et al*., 2013a). Additionally, the number of sperm subpopulations and the percentages of sperm belonging to each subpopulation could vary not only between species but also when semen samples are submitted to different procedures, such as centrifugation and sperm selection techniques (Martinez-Alborcia *et al*., 2013; Stoll *et al*., 2013; Urbano *et al*., 2013; Anel-López *et al*., 2015). Although standard and colloid centrifugation have shown promising results in improving conventional donkey sperm quality parameters, such as total and progressive sperm motility, plasma membrane integrity or normal sperm morphology (Ortiz *et al*., 2015a), the effect of these treatments on donkey sperm subpopulations remains unknown.

In sperm selection studies, the distribution of the sperm subpopulations or the percentages of a certain parameter prior to and after centrifugation are compared (Johannisson *et al*., 2009; Cabrera *et al*., 2014; Hidalgo *et al*., 2017; Morrell *et al*., 2017). In this sense, the total amount of sperm cells recovered are evaluated (Morrell *et al*., 2014), but not the amount of sperm belonging to each subpopulation. Moreover, motile sperm subpopulation analysis works with changes on percentages, and includes only motile sperm (Martinez-Pastor *et al*., 2005; Flores *et al*., 2008; Muiño *et al*., 2009; Ortega-Ferrusola *et al*., 2009; Dorado *et al*., 2010; Dorado *et al*., 2013a), excluding immotile sperm from the analysis. In this sense, a new approach to evaluate sperm subpopulations would allow an understanding of the changes in sperm subpopulations arising from different preparation techniques from a more practical point of view.

The aim of this study was, therefore, to compare the effect of three centrifugation procedures after thawing on motile sperm subpopulations, assessed by a new analysis that integrates immotile sperm and recovery rate.

## Materials and methods

### Semen collection and evaluation

All animal procedures were performed in accordance with the Spanish laws for animal welfare and experimentation, under the supervision of the Bioethical and Biosafety Committee of the University of Cordoba. Semen was collected from four clinically healthy Andalusian donkeys (aged 6-15 years) using an artificial vagina in the presence of a jenny in estrus. Animals were housed in individual paddocks placed at the Veterinary Teaching Hospital of the University of Cordoba, Spain. Semen was collected from each animal twice per week on different sampling occasions, obtaining twelve ejaculates in total (three ejaculates per animal). Fresh semen samples were analysed prior to freezing and had a gel-free volume of at least 44.0 ml, sperm concentration > 208.7 x10^6^ sperm/ml, total sperm motility > 90.3%, progressive sperm motility > 67.3% and normal sperm morphology > 72.7% evaluated as described by Ortiz *et al*. (2014).

An aliquot from each ejaculate was diluted with a skimmed milk extender (INRA 96, IMV Technologies, L'Aigle, France) to reach a sperm concentration of approximately 25 x10^6^ spermatozoa per ml, incubated at 37ºC for 10 min and then assessed for sperm motility as described later.

### Semen freezing and thawing

Semen samples were frozen following a protocol previously used for donkeys (Ortiz *et al*., 2015b). Briefly, fresh semen was diluted in a ratio 1:1 (v:v) with EquiPro (Minitüb, Tiefenbach, Germany) and then centrifuged 7 min at 400 x*g*. The sperm pellet was re-extended in semen freezing medium with glycerol (Gent, Minitüb, Tiefenbach, Germany) to a final concentration of 200 x10^6^ sperm/ml. Sperm were slowly cooled to 5ºC for 2 h into an Equitainer and then loaded in 0.5 ml plastic straws. The straws were frozen horizontally in racks placed 2.5 cm above the surface of liquid nitrogen (LN_2_) for 5 min and placed into LN_2_ tanks. Straws were thawed by immersion in a 37ºC water bath for 30 s.

### Computer-Assisted Sperm Motility Analysis (CASA)

Sperm motility was evaluated in fresh semen and in frozen-thawed semen samples by CASA (Sperm Class Analyzer, SCA v5.01, Microptic S.L., Barcelona, Spain). The features of this system have been described previously (Ortiz *et al*., 2014). Sperm kinematics were evaluated for a minimum of 200 sperm per sample in three drops, using two randomly chosen microscopic fields per drop. The kinematics evaluated were total (TM, %) and progressive motility (PM, %), curvilinear (VCL, μm/s), straight line (VSL, μm/s) and average path velocities (VAP, μm/s), linearity (LIN, VSL/VCLx100), straightness (STR, VSL/VAPx100), wobble (WOB, VAP/VCLx100), amplitude of lateral head displacement (ALH, μm) and beat cross frequency (BCF, Hz).

### Post-thaw sperm procedures

Four aliquots of each frozen-thawed semen sample were processed according to the following procedures:

At least two different straws were thawed per treatment. The uncentrifuged diluted control (UDC) was thawed and directly diluted with INRA 96 to a final concentration of 25 x10^6^ sperm/ml. Post-thaw sperm parameters were analysed as described above. Sperm washing (SW) was performed immediately after thawing; one semen straw was extended 1:1 in INRA 96 and then centrifuged at 400 *g* for 7 min. The supernatant was removed and the sperm pellet was resuspended as described for UDC. Single layer centrifugation (SLC) was performed at 300g for 20 min using two colloids based on silane-coated silica particles: EquiPure (SLC-E, EquiPure^TM^ bottom layer, Nidacon International AB, Gothenburg, Sweden) or AndroColl-Equine (SLC-A, Minitüb, Tiefenbach, Germany), following the methodologies described by Ortiz *et al*. (2015a) and Ortiz *et al*. (2015b), respectively. The resulting pellets of SLC-E and SLC-A were resuspended as in UDC and SW.

### Recovery rate of total, motile and progressive sperm

The percentage of recovered sperm after each centrifugation procedure was calculated according to the following formula:

Recovery rate = (number of sperm* in pellet/number of sperm* in initial load) x 100

*Sperm recovery rate was separately calculated for: total, motile and progressively motile sperm

### Recovery rate of sperm subpopulation (sP)

The recovery rate of the sperm subpopulation more related with fertility in donkeys (Dorado *et al*., 2013a), the most vigorous and progressive spermatozoa (sP4), was calculated as follows:

*Recovery rate of sP4 = (Total sperm recovery of the centrifugation treatment x TM after centrifugation x sP4 after centrifugation / TM prior to centrifugation x sP4 prior to centrifugation)*

### Statistical analysis

Data were arcsin transformed prior to statistical analysis. Results were expressed as mean ± standard deviation (SD). Differences between mean values of fresh and frozen thawed sperm motility and post-thaw sperm recovery rate were analyzed using a general linear model (PROC GLM) with animals, treatments and ejaculates as fixed effect. The Duncan test was used for *post hoc* analyses. Secondly, sperm subpopulations analysis was performed over a data matrix of 32,993 individual motile spermatozoa from all the semen samples obtained and processed with the different treatments performed. A principal component analysis (PRINCOMP) followed by the FASTCLUS clustering procedure was used to classify the spermatozoa of the data set into a reduced number of subpopulations according to their patterns of movement as previously described (Martinez-Pastor *et al*., 2005). The summary statistics of the relative frequencies of spermatozoa belonging to each subpopulation were calculated and compared by ANOVA and chi-squared test (FREQ procedure). The motility kinematics of the sperm belonging to each subopulation were compared by ANOVA. Finally, the relative frequency distribution of the motile sperm subpopulations identified were assessed between and within treatments. All analyses were performed with SAS statistic package v9.0 (SAS Institute Inc., Cary, NC, USA). The level of significance was set at P < 0.05.

## Results

In general, TM and PM were significantly higher (P < 0.001) in fresh semen (96.25 ± 2.98 and 83.04 ± 6.51) in comparison to UDC frozen-thawed semen samples (64.81 ± 17.09 and 51.62 ± 16.14), respectively. Additionally, colloid single layer centrifugation resulted in higher sperm parameters in comparison to SW and UDC. SLC-A obtained similar values for PM (77.21 ± 17.08) in comparison to fresh semen. In addition, most of the sperm kinematics were significantly higher in SLC-A in comparison to the other post-thaw procedures obtaining values similar to (VCL), or even higher than (VSL, VAP, LIN, STR and WOB) fresh semen (Tab. 1).

**Table 1:** Comparison of sperm parameters assessed before freezing (fresh semen) and after different procedures performed after thawing.

Sperm parameters	Fresh semen	Post-thaw sperm processing			
		UDC	SW	SLC-E	SLC-A
TM (%)	96.25 ± 2.98^a^	64.81 ± 17.09^c^	60.81 ± 18.00^c^	83.49 ± 13.89^b^	83.90 ± 14.64^b^
PM (%)	83.04 ± 6.51^a^	51.62 ± 16.14^c^	50.91 ± 18.20^c^	70.83 ±19.34^b^	77.21 ± 17.08^ab^
VCL (μm/s)	169.72 ± 24.30^a^	137.89 ± 16.07^c^	155.99 ± 18.75^ab^	140.23 ± 28.80^bc^	165.14 ± 22.98^a^
VSL (μm/s)	107.09 ± 9.64^b^	111.00 ± 11.22^b^	132.41 ± 15.52^a^	107.77 ± 21.54^b^	140.61 ± 20.16^a^
VAP (μm/s)	140.72 ± 18.97^bc^	127.59 ± 14.69^c^	146.33 ± 18.71^ab^	126.82 ± 25.70^c^	155.70 ± 22.83^a^
LIN (%)	59.86 ± 4.48^d^	70.31 ± 3.74^c^	76.58 ± 2.91^b^	72.30 ± 5.84^c^	80.67 ± 3.40^a^
STR (%)	72.08 ± 5.73^d^	78.49 ± 2.31^c^	83.72 ± 2.28^b^	80.72 ± 4.75^bc^	87.13 ± 2.64^a^
WOB (%)	80.06 ± 3.60^d^	84.91 ± 2.31^c^	87.68 ± 2.47^b^	86.46 ± 2.76^bc^	90.40 ± 2.64^a^
ALH (μm)	4.16 ± 0.92^a^	2.37 ± 0.34^b^	2.44 ± 0.21^b^	2.73 ± 0.58^b^	2.65 ± 0.37^b^
BCF (Hz)	7.69 ± 1.46^b^	8.23 ± 0.81^b^	8.13 ± 0.63^b^	9.64 ± 1.14^a^	9.30 ± 0.74^a^

Different letters in the same row indicate significant differences between treatments (P < 0.001). Values are expressed as mean ± standard deviation. UDC = Uncentrifuged diluted control; SW = Sperm washing; SLC-E/A = Single layer centrifugation using Equipure/Androcoll; TM = Total motility; PM = Progressive motility; VCL = Curvilinear velocity; VSL = Straight-line velocity; VAP = Average path velocity; LIN = Linearity; STR = Straightness; WOB = Wobble; ALH = Amplitude of lateral head displacement; BCF = Beat-cross frequency.

The principal component analysis followed by the cluster procedure identified four motile subpopulations. Summary statistics for the motility characteristics of each subpopulation are shown in Table 2. Subpopulation 1 (sP1) included spermatozoa with relatively low velocity (medium VCL, VSL and VAP) but with high progressiveness (high LIN, STR, WOB, BCF and low ALH), recovering 32.2% of the total motile population. Subpopulation 2 (sP2) comprised 11.1% of sperm population, including highly active but non-progressive spermatozoa (high VCL and ALH, low LIN and STR and moderate BCF). Subpopulation 3 (sP3) contained the lowest number of spermatozoa (9.5%) and included spermatozoa whose movements were less vigorous (low VCL, VAP, ALH and BCF) and less progressive (low VSL, LIN and STR). Subpopulation 4 (sP4) contained the largest number of spermatozoa (51.5%), which were the most vigorous spermatozoa (highest VCL and BCF and high ALH) and progressive (highest VSL and VAP).

**Table 2:** Motility parameters for the four sperm subpopulations (sP1, sP2, sP3, and sP4) defined after analysis of the entire set of semen samples.

Subpopulation	n (%)	Sperm motility patterns							
		VCL (μm/s)	VSL (μm/s)	VAP (μm/s)	LIN (%)	STR (%)	WOB (%)	ALH (μm)	BCF (Hz)
sP1	10632 (32.2)	128.12 ± 36.71^c^	102.85 ± 32.15^b^	117.09 ± 34.26^c^	81.18 ± 14.85^b^	88.61 ± 12.95^b^	91.30 ± 6.87^b^	2.51 ± 0.98^d^	8.72 ± 3.34^b^
sP2	3658 (11.1)	201.81 ± 28.47^b^	80.66 ± 30.81^c^	158.93 ± 31.56^b^	39.30 ± 12.90^c^	51.41 ± 19.28^d^	78.35 ± 8.50^c^	5.31 ± 1.08^a^	7.75 ± 3.04^c^
sP3	3150 (9.5)	77.73 ± 44.47^d^	30.86 ± 21.24^d^	57.77 ± 39.63^d^	39.09 ± 13.75^c^	56.40 ± 19.53^c^	70.76 ± 13.76^d^	2.75 ± 1.05^c^	5.50 ± 2.91^d^
sP4	15553 (47.1)	208.38 ± 26.28^a^	177.31 ± 32.31^a^	194.02 ± 26.78^a^	85.31 ± 12.13^a^	91.34 ± 9.99^a^	93.14 ± 5.48^a^	3.28 ± 1.18^b^	10.18 ± 3.51^a^

n = number of spermatozoa. Values are expressed as mean ± standard deviation. Different superscript letters (a-d) in the same column indicate significant differences (P < 0.001) between subpopulations. Abbreviations: VCL= curvilinear velocity; VSL = straight-line velocity; VAP = average path velocity; LIN = linearity; STR = straightness; WOB = wobble; ALH = amplitude of lateral head displacement; BCF = beat-cross frequency.

**Figure 1:**
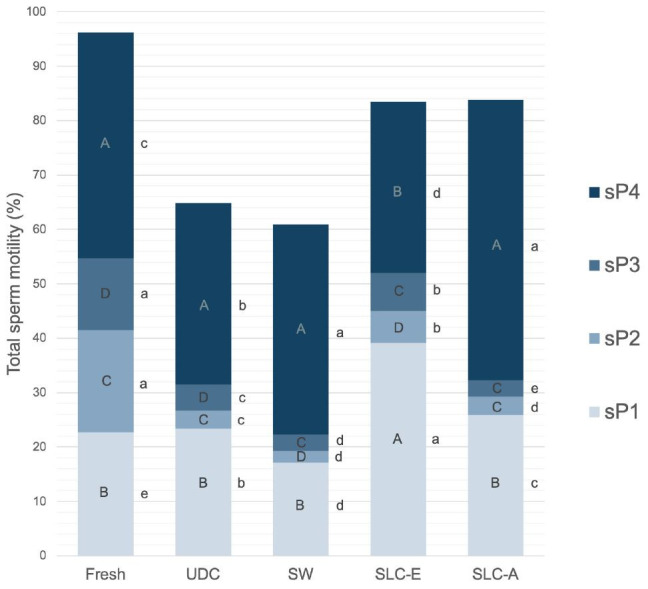
Relative frequency distribution of motile sperm subpopulations (sP1-sP4) between and within treatments. Different letters (A-D) inside columns indicate significant differences within treatments (P < 0.01). Different letters (a-e) beside bars indicate significant differences between treatments (P < 0.05).

There were significant differences in the distribution of the four sperm subpopulations between (P < 0.05, letters a-e) and within (P < 0.01, letters A-D) treatments (Fig. 1). Sp4 was the most representative subpopulation in all treatments except for SLC-E where sP1 was the largest. Additionally, the percentage of sperm belonging to sP4 was higher in SLC-A and SW treatments followed by UDC, fresh semen and SLC-E, respectively.

**Figure 2:**
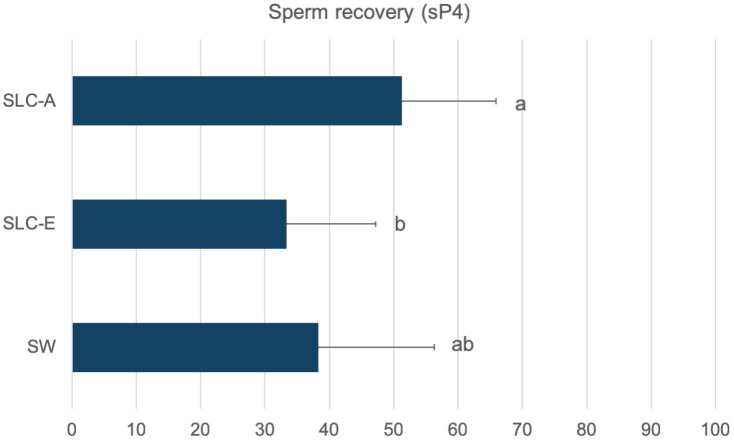
Sperm recovery rate of subpopulation 4 (sP4, %) after centrifugation by sperm washing (SW) and colloid centrifugation (SLC-E, SLC-A). Different letters indicate significant differences among between treatments (P < 0.05).

In general, sperm recovery was similar between SLC-E (30.31%) and SLC-A (28.15%) but both were lower than SW (60.05%) (Tab. 3). However, the recovery of sperm belonging to sP4, was higher for SLC-A (51.27%) in comparison to SLC-E (33.32%), obtaining SW (38.26%) a similar value to both SLC-A and SLC-E treatments (Fig. 2).

**Table 3:** Sperm recovery rate (%) obtained in the pellets after different post-thaw sperm procedures.

Treatments	Recovery rate of		
	Total sperm	Total motile sperm	Progressively motile sperm
Sperm Washing	60.05 ± 15.37^a^	61.20 ± 14.94^a^	63.18 ± 17.05^a^
SLC-E	30.31 ± 10.39^b^	41.02 ± 15.62^b^	42.56 ± 17.44^b^
SLC-A	28.15 ± 14.08^b^	36.71 ± 15.12^b^	44.74 ± 20.05^b^
P value	P < 0.001	P < 0.001	P < 0.05

Values are expressed as mean ± standard deviation. SLC = Single layer centrifugation.

## Discussion

This study compared three centrifugation protocols for sperm motility improvement assessed by both a traditional analysis and a new analysis. According to our results, SLC using each colloid increased TM and PM compared to SW and UDC, which is in agreement with other studies performed in donkey semen (Ortiz *et al*., 2015a; Ortiz *et al*., 2015b). Previous studies have widely shown the impact of SLC in other parameters in stallions and donkeys, such as sperm viability, sperm DNA fragmentation, reactive oxygen species and fertilizing ability (Lindahl *et al*., 2012; Morrell *et al*., 2013; Gamboa *et al*., 2017; Ortiz *et al*., 2017). Regarding the sperm motility analysis, it is not only motility per se that is important but also the pattern (kinematics) of this movement. Both SLC methods significantly increased ALH and BCF, which are related to sperm vigour (Cancel *et al*., 2000). Moreover, VCL, VSL and, VAP, which indicate rapidness, are correlated with fertility rates in several mammalian species (Olds-Clarke, 1996; Robayo *et al*., 2008; Macías García *et al*., 2009b). Surprisingly, the highest values of these velocities were shown after SLC-A, being even greater than those obtained in fresh semen. These results confirm and extend to velocity parameters the fact that SLC through a colloidal matrix can select a population of spermatozoa that is positively enhanced in most of the sperm quality parameters usually assessed (Ortiz *et al*., 2015a). On the contrary, SLC-E did not achieve such values and they were lower than SW. Seminal plasma could explain differences in the sperm motility patterns among fresh and frozen-thawed procedures since most of them is removed before cryopreservation. However, a recent study has shown that motility parameters were not affected by adding seminal plasma to thawed stallion sperm and cryoinjuries were not repaired (Al-Essawe *et al*., 2018a). Moreover, seminal plasma added to SLC-selected stallion sperm after thawing showed a decrease in binding ability, using a heterologous zona binding assay with *in vitro* matured bovine oocytes, in comparison to seminal plasma-free sperm cells (Al-Essawe *et al*., 2018b). Further studies are needed to clarify this issue. According to mean values analysis obtained in this study, SLC-A selected the most rapid, progressive and vigorous spermatozoa.

Our study revealed four sperm subpopulations as previously described in fresh, cooled and frozen-thawed donkey semen (Flores *et al*., 2008; Miró *et al*., 2009). In agreement with these studies, cryopreservation had a significant effect on the distribution of these subpopulations. According to the results obtained, it seems clear that sperm subpopulations with non-progressive movement (sP2 and sP3) are not able to survive freezing and thawing and become immotile (Flores *et al*., 2008). When thawed sperm were submitted to sperm washing, the proportions of sP1 (low velocity and high progressiveness), sP2 (high activity but non-progressiveness) and sP3 (less vigour and less progressiveness) were decreased and sP4 (highest vigour and progressivity) was increased. It seems that fast and progressive sperm can withstand the stress caused by centrifugation and they pass more easily into the pellet.

Regarding SLC-E, the subpopulation with a higher number of spermatozoa was sP1. This could be due to the fact that the specific formulation of this colloid applied to Donkey sperm seems to select motile and progressive sperm, regardless of their velocity. After SLC-A, the highest number of sperm were in sP4. Thus, either this colloid selects fast and progressively motile sperm (sP4) better than SLC-E or it has a less deleterious effect on sperm motility than SLC-E. In stallions, SLC resulted in selection of progressively motile spermatozoa from frozen-thawed semen with relatively low velocity (similar to sP1 in this study) (Macías García *et al*., 2009a). In dogs, samples centrifuged with Androcoll-C and PureSperm 40/80 had an increased subpopulation with high speed and progressive motility (similar to sP4 in this study) (Dorado *et al*., 2013b). Our results cannot be compared with other studies in donkey semen since, to the best of the author’s knowledge, this is the first study which assesses the effect of colloid centrifugation on motility in donkey sperm subpopulations. The subpopulation of rapid and highly progressive spermatozoa, which correspond to sP4 in this study, has been found in a higher proportion in fresh ejaculates of donkeys with better fertility rates (Dorado *et al*., 2013a). However, it is not possible to assume that a higher proportion of sP4 in SLC-A samples is responsible for higher pregnancy rates without fertility trials.

Although the study of the motile subpopulations allows changes in motile sperm distribution to be evaluated accurately, sometimes the results can be confusing. In this study, both immotile sperm and the recovery rate of sP4 have been included for a better understanding of motile subpopulation analysis. On the one hand, immotile sperm allows the visualization of the total number of sperm analysed between and within treatments, including those will not likely fertilize. On the other hand, the recovered sperm belonging to sP4 after centrifugation offers a new approach of sP analysis, showing the amount of the most vigorous and progressive sperm available in the sample (sP4), those will likely fertilize. The subpopulation sP4 is not present in subfertile donkeys ejaculates, as it has been described in previous studies (Dorado *et al*., 2013a). Surprisingly, when assessing the sperm recovery of sP4 we found no differences between the available sP4 sperm in SLC-A (apparently the best treatment according to traditional analysis) and SW. From a practical point of view, those recovery rates meant that sperm washing, the standard centrifugation procedure for equine semen processing, recovered the same amount of fast and progressive sperm as colloid centrifugation, apparently the best treatment according to traditional analysis. Pregnancy rates in Jennies ranged from 28 to 36% after artificial insemination (AI) with 500-1000 x10^6^ frozen-thawed sperm (Rota *et al*., 2012; Oliveira *et al*., 2016). Both, SLC and SW procedures offer at least this amount of vigorous and progressive sperm available for AI. Further studies are needed to compare pregnancy rates among all these treatments as previously indicated.

In conclusion, samples processed by SLC have higher sperm motility percentages than SW but, after integrating sperm motile subpopulation analysis and available motile sperm after each technique in the comparison, they are equally efficient in recovering sperm from the most vigorous, fast and progressive motile subpopulation (sP4).
